# Assessment of the quality of nursing work life and its related factors among critical care nurses

**DOI:** 10.3389/fpubh.2024.1305686

**Published:** 2024-02-07

**Authors:** Majdi M. Alzoubi, Shaimaa Al-momani, Khalid Al-Mugheed, Islam Owiedat, Ghada Mohammad Abu Shosha, Amany Anwar Saeed Alabdullah, Samira Ahmed Alsenany, Sally Mohammed Farghaly Abdelaliem

**Affiliations:** ^1^Faculty of Nursing, Al-Zaytoonah University of Jordan, Amman, Jordan; ^2^College of Nursing, Riyadh Elm University, Riyadh, Saudi Arabia; ^3^Faculty of Nursing, Zarqa University, Zarqa, Jordan; ^4^Department of Maternity and Child Health Nursing, College of Nursing, Princess Nourah bint Abdulrahman University, Riyadh, Saudi Arabia; ^5^Public Health Department, Faculty of Nursing, King Abdulaziz University, Jeddah, Saudi Arabia; ^6^Department of Nursing Management and Education, College of Nursing, Princess Nourah bint Abdulrahman University, Riyadh, Saudi Arabia

**Keywords:** critical care nurses, quality of work, quality of sleep, workplace noise, Jordan, nursing, factors

## Abstract

**Background:**

Quality of work life (QWL) refers to the degree to which employees contribute to the organization’s goals while also experiencing personal and professional satisfaction. This study conducted to evaluate the quality of nursing work life (QNWL) level and its associated factors among nurses working in critical care units.

**Methods:**

A convenience sample technique among critical care nurses in Jordan by using a cross-sectional, descriptive design. A self-reported questionnaire was used. A Pittsburgh Sleep Quality Index (PSQI), and the Andersson and Lindgren questionnaires scale were used in data collection.

**Results:**

The total mean scores of QNWL were *M* = 86.17 (SD = 35.12), which is slightly below the expected middle value (87.5). The nurses have a higher psychological relation, *M* = 18.28 (SD = 8.99), whereas they have the lowest competence development, *M* = 11.44 (SD = 5.56). There was statistical significance between workplace noise, and workplace sources of noise, quality of sleep and QNWL.

**Conclusion:**

The outcomes also highlighted the significance of undertaking additional interventional research studies in the future in order to identify practical strategies to improve nurses QNWL. As a result, the nursing care given to the patients and their families may be improved.

## Introduction

Quality of work life (QWL) is now recognized as a significant issue, and numerous studies have been conducted on the subject (1, 2). These studies explore the relationship between QWL and various organizational outcomes, and found significant relationship between job performance and quality of working life in all the aspects. QWL refers to the degree to which employees contribute to the organization’s goals while also experiencing personal and professional satisfaction (3). The nurses make up the largest and most diverse segment of the healthcare workforce (2, 4, 5). Nurses’ QWL is the level to which licensed nurses are able to meet significant personal demands as a result of their experiences at work while meeting organizational standards (6). The idea of worker satisfaction is increasingly crucial since workers feel at ease in environments where they must be appreciated, valued, and recognized (4, 7, 8).

The quality of nursing work life (QNWL) comprises factors like job security, job description, nursing development plans as measurements, relationships with coworkers, wages, and job satisfaction (9). Previous studies have demonstrated the significance of QNWL assessment (10, 11). Nevertheless, some studies have found that nurses have high levels of job dissatisfaction, burnout, and resignation intent (11, 12) and enhancing their QWL has become a difficult challenge in the healthcare system since the 1970s (13). One of the issues that influences the provision of the best nursing care is the need to keep and recruit nurses (14). It’s essential for healthcare organizations to address the specific challenges and concerns that nurses may be facing. Improving QNWL can lead to better job satisfaction, increased retention rates, and enhanced patient care.

Increasing nursing heavy workload and widespread shortages are now major global problems (15–17). In Jordan the majority of nurses reported that there were not enough nurses in the workplace and that their task was heavy. As a result, nurses failed to provide patients with high-quality care since they were not satisfied with their jobs (18). Despite receiving education and training to provide patients with high-quality nursing care, nurses’ working environments and interference with their personal needs remain neglected (19). According to a recent scoping review study (20), improvements to the working environment conditions at all management levels should be made. Therefore, it should be taken into account to perform regular evaluations and assessments for factors impacting nurses’ QWL and the work environment.

Many studies have explored many factors that influence QNWL, including sociodemographic and work-related variables (21–24). The most important sociodemographic characteristics associated with QNWL were gender, educational attainment, and marital status (2, 5, 21, 25). On the other side, QNWL is influenced by aspects relating to the workplace, such as attitude, opportunity, job characteristics, stress levels, career possibilities, difficulties faced, room for growth and development, risks involved, and rewards (4, 26, 27). An earlier study in Jordan found no significant relationship between QNWL scores and all the sociodemographic and work-related variables (18). However, the fact that it was the only study that investigated QNWL in Jordan indicates that more research is necessary. The current study’s objective is to evaluate the QNWL level and its associated factors among nurses working in critical care units (CCUs) in Jordan.

## Method

### Research design

A cross-sectional descriptive approach was employed to address the study’s objective. The current study was carried out in CCUs at both public and private hospitals in Amman, Jordan. Jordan’s public and private healthcare facilities provided medical care for 24 h a day, throughout the week.

### Study population and sampling

The participants in the study were registered nurses (RN) employed in Jordan’s CCUs. All registered nurses working at CCUs in government, private, and academic hospitals in Jordan were included in the current study. Data from CCUs at the chosen hospital was collected using a non-probability convenience sampling technique. Using the G*Power 3.1.10 program, the sample size was calculated. A minimum sample size of 186 was required for the regression test (power = 0.90, =0.05, and effect size = 0.08 with 10 predictors). The inclusion criteria included RN nurses that work a rotatable 8-h shift, have at least 1 year of prior work experience, and are willing to participate. Nurses working in critical care who had sleep apnoea were not included.

### Instruments

Self-reported questionnaires were used to assess the demographic characteristics, work-related factors, subjective sleep quality, and perceived quality of nursing work life (QNWL) of the participants. The demographic information covered the nurses’ age in years, gender, marital status, BMI, level of education, and smoking habits. The work-related factors include shift-able work, shift type, and workplace noise. The sleep quality of the nurses was evaluated using the Arabic version of the Pittsburgh Sleep Quality Index (PSQI) (28). The PSQI is a collection of 19 self-reported questions that evaluate different aspects of sleep quality. Higher scores indicate poor sleep quality. Additionally, the QNWL was evaluated using the Andersson and Lindgren self-reported questionnaire (29). The questionnaire has 35 items divided into six subscales: Psychosocial relations, Commitment, Work satisfaction, openness or closeness, Competence development, and security or insecurity. Each item is graded on a 5-point Likert scale ranging from 1 (strongly disagree) to 5 (strongly agree). The item total scores vary from 35 to 175. Higher ratings showed that nurses had a favorable impression of the QNWL. The mean was used for scoring the total scale as follows: 1–1.79 = poor, 1.80–2.59 = fair, 2.6–3.39 = good, 3.40–4.19 = very good, and 4.20–5.0 = high (30). Cronbach’s alpha coefficient of the scale is 0.93 (30). The Arabic version of this instrument was administered in the current study, and it has good validity and reliability, with a Cronbach’s alpha of 0.940 (31).

### Data collection

The first step was to contact and inform the chosen hospitals of the study. To obtain a list of nurses who satisfied the study’s inclusion criteria, the targeted CCU nurse heads were contacted. The aim, significance, benefits, and risks of the study were explained to prospective volunteers. Nurses who volunteered to participate were given the questionnaires by the researcher during their break in the restroom. The survey was given to the participants with the option to either complete it or give it back to the researcher or to bring it the following day between October and November 2022.

### Ethical considerations

The Jordanian Al-Zaytoonah University gave the approval for the study with IRB No. (1269312080). Afterward, Jordan’s Al-Zaytoonah University sent a cover letter describing the study’s objectives to the administrators of the selected institutions. It was made clear to the participants that they are free to withdraw from the study at any moment and that they should participate voluntarily. In addition, it was made clear to the participants that there would be no financial compensation for their participation. When participants fill out and return surveys, it is considered that they consent to participate. All of the data, including the names of the participants, was kept private by the researchers as well.

### Data analysis

Descriptive analysis and binary logistic regression analysis were the statistical analyses performed in the current study. The percentages, frequency, standard deviation, and mean are all obtained in the descriptive analysis. The logistic regression analysis was performed to identify significant factors associated with QNWL (i.e., good, very good, and high) among Jordanian CCU nurses. In the logistic regression analysis, simple logistic regression was initially performed to obtain the crude odds ratio (COR), and those that had a value of *p* 0.05 were considered important predictors of QNWL and included in the multiple logistic regression to obtain their adjusted odds ratio (AOR).

## Results

The descriptive characteristics of the study participants are shown in Table 1. The study involved 250 nurses, with a mean age of 33.14 (SD = 4.61) and a mean BMI of 2.49 (SD = 0.58). In this study, there are exactly as many males as females (125 males and 125 females). Of the total participants, 149 (59.6%) were married, and 144 (57.6%) were nonsmokers. The majority of participants (88.0%, *n* = 220) have a bachelor’s degree and work shifts (89.2%, *n* = 223). More than half of the participants (52.8%, *n* = 132) worked an 8-h shift. The majority of the participants (75.6%, *n* = 189) reported being exposed to occupational noise, with equipment being the most commonly reported source of noise (28.4%, *n* = 71). Additionally, the majority of the nurses (31.2%, *n* = 78) reported fair QNWL.

**Table 1:** Sociodemographic and work-related characteristics of the study participants.

	**Total (*n* = 250)**	
**Variable**	***F* (%)**	**Mean (SD)**
**Age**		33.1 (4.61)
**BMI**		2.5 (0.58)
**Gender**		
Male	125 (50.0)	
Female	125 (50.0)	
**Marital status**		
Single	80 (32.0)	
Married	149 (59.6)	
Divorced	21 (8.4)	
**Smoking status**		
Yes	144 (57.6)	
No	106 (42.4)	
**Educational level**		
Bachelor	220 (88.0)	
Master	27 (10.8)	
Others	3 (1.2)	
**Shiftable work**		
Yes	223 (89.2)	
No	27 (10.8)	
**Type of shift**		
None	23 (9.2)	
8 h	132 (52.8)	
12 h	80 (32.0)	
Other	15 (6.0)	
**Workplace noise**		
Yes	189 (75.6)	
No	61 (24.4)	
**Workplace source of noise**		
None	61 (24.4)	
Increased mortality in units	66 (26.4)	
Visitors	52 (20.8)	
Equipment	71 (28.4)	
**QNWL group**		
Poor	77 (30.8)	
Fair	78 (31.2)	
Good	31 (12.4)	
Very good	53 (21.2)	
High	11 (4.4)	

Table 2 presents the mean score of the QNWL and all six components. In this study, the total mean scores of QNWL were *M* = 86.17 (SD = 35.12), which is slightly below the expected middle value (87.5). The nurses have a higher psychological relation, *M* = 18.28 (SD = 8.99), whereas they have the lowest competence development, *M* = 11.44 (SD = 5.56).

**Table 2:** Total and components scores of the QNWL.

**QNWL components**	**Total possible scores**	**Mean (SD)**	**Range in the sample**
Overall quality of nursing care	35–175	86.17 (35.12)	35–160
Psychological relation	8–40	18.24 (8.99)	8–37
Commitment	5–25	13.42 (6.09)	5–25
Work satisfaction	6–30	13.73 (6.42)	6–28
Openness and closeness	5–25	13.96 (4.21)	5–24
Competence development	5–25	11.44 (5.56)	5–23
Security/insecurity	6–30	13.74 (7.63)	6–28

Table 3 shows that three variables (i.e., workplace noise, workplace source of noise, and sleep quality) were retained in the final model and therefore considered significant predictors of QNWL. For workplace noise, those who reported no were 3.7 times more likely to have a good QNWL than those who reported yes (AOR = 3.67, *p* = 0.005). For the source of workplace noise, the increase in mortality in units’ sources was 67% less likely to have good QNWL than the equipment source (AOR = 0.33, *p* = 0.021), and visitors sources were 53% less likely to have good QNWL than the equipment source (AOR = 0.47, *p* = 0.171). Those with good quality sleep were about 25 times more likely to have good QNWL than those with poor sleep quality (AOR = 25.31, *p* < 0.001).

**Table 3:** Factors associated with good QNWL among Jordanian critical care nurses.

**Variable**	**COR (95% CI)**	***P***	**AOR (95% CI)**	***P***
**Age**	0.95 (0.89, 1.01)	0.103	–	–
**Gender**				
Male	1.12 (0.65, 1.94)	0.677	–	–
Female	1			
**Marital status**				
Single	5.40 (1.17, 24.86)	0.030	–	–
Married	3.73 (0.83, 16.71)	0.086	–	–
Divorced	1			
**BMI**				
Healthy	2.15 (0.45, 10.38)	0.339	–	–
Overweight	1.58 (0.32, 7.80)	0.574	–	–
Obese	1			
**Smoking status**				
Yes	0.58 (0.33, 0.99)	0.048	–	–
No	1			
**Educational level**				
Bachelor	–		–	–
Master	–		–	–
Others	–		–	–
**Shift able work**				
Yes	1.50 (0.58, 3.89)	0.401	–	–
No	1			
**Type of shift**				
None	0.42 (0.09, 1.93)	0.265	–	–
8 h	1.03 (0.33, 3.21)	0.953	–	–
12 h	0.62 (0.19, 2.05)	0.436	–	–
Other	1			
**Workplace noise**				
Yes	5.92 (3.17, 11.04)	< 0.001	3.67 (1.49, 9.03)	0.005
No	1		1	
**Workplace source of noise**				
None	3.67 (1.78, 7.59)	<0.001	–	–
Increased mortality in units	0.46 (0.20, 1.06)	0.069	0.33 (0.13, 0.84)	0.021
Visitors	0.40 (0.15, 1.03)	0.056	0.47 (0.16, 1.38)	0.171
Equipment	1		1	
**Quality of sleep**				
Good	22.87 (8.78, 59.61)	<0.001	25.31 (9.22, 69.51)	<0.001
Poor	1		1	

COR, crudes odds ratio; AOR, adjusted odds ratio; CI, confidence interval.

## Discussion

The present study aimed to investigate the quality of nursing work life (QNWL) and its related factors in public, private, and educational hospitals in Jordan. One of the essential findings of the present study was that the majority of the nurses (31.2%) reported fair QNWL, with only a few (4.4%) reporting high QNWL. This is consistent with the findings of a previous study among Iranian nurses, which showed that 61.4% of participants believed that their QNWL was at a low-to-moderate level and only 3.6% reported their QNWL to be high (32). However, a previous study among Jordanian nurses revealed that the participants had moderate QNWL 18. Furthermore, a number of studies have produced contradictory findings about nurses’ QWL. For instance, Lee, Dai, stated that nurses in Taiwanese hospitals had an average level of QNWL (33). On the other side, Hesam, Asayesh study findings showed that the majority of nurses working in hospitals at Gorgan University of Medical Sciences had higher-than-average and desirable levels of QNWL satisfaction (34). In contrast another research in Saudi Arabia, revealed that the majority of nurses had poor QNWL balance (35). The varying working environments in various hospitals may be one of the possible sources of these contradictory findings (5, 36).

Nurses in the current study had mean values for the components of psychological relationships, work satisfaction, competence development, and security or insecurity that were all slightly below the expected middle values. This indicates that the nurses possess fair QNWL regarding these components. According to the earlier research conducted by Suleiman, among Jordian nurses, nurses claimed that their workload was excessive and that there were not enough nurses in the workplace when it came to the work design component (18). The majority of nurses believed they did not deliver high-quality patient care and were not satisfied with their work. These findings were supported by previous studies (5, 21, 26). In addition, earlier research revealed that few nurses felt appreciated by senior management and were able to participate in decisions, which is similar to the findings of the current study (21, 37). Furthermore, Almalki, FitzGerald, reported that the skill mix in nurse work environments was frequently insufficient and that there were few prospects for career progression (32).

With regards to the other components of QNWL, including commitment, openness, and closeness, the nurses had mean values of QNWL above the middle value. This demonstrates the strong devotion to their jobs and good working relationships among the nurses in the current study. According to another study, the organizational commitment of nurses was generally high, with just 18.9% of respondents rating it as low (32). According to researchers, organizational commitment is a multifaceted factor that promotes productivity (38). The results of the earlier studies reported that nurses’ productivity was at an average level (32, 39). Additionally, prior studies have shown that nurses experience a sense of belonging at work, work in a team, feel appreciated by doctors, can connect with the other therapists on the unit, and receive feedback from the nurse management regarding their performance (18, 26).

There was not a statistically significant association between the QNWL and all of the sociodemographic variables in the current study. The findings are in line with those of an earlier study among Jordanian nurses that discovered no statistically significant associations between QNWL and sociodemographic factors such as gender, marital status, and level of education (18). However, some studies have found that age and marital status can significantly influence QNWL (2, 40).

The results of the current study revealed that workplace noise, workplace source of noise, and quality of sleep had a significant effect on QNWL. The most important predictor of good QNWL is sleep quality. These results indicate that better sleep quality and a quiet working environment promote better QNWL. According to the findings of a Momeni, Shafipour, nurses with better sleep quality had higher mean QNWL ratings than nurses with poorer sleep quality (41). Their findings indicate a significant association between QNWL and sleep quality, with better QNWL associated with better sleep. Furthermore, research studies indicate an association between workplace hazards, such as noise, ergonomics, and stress, and hospital employees’ perceptions of a low or moderate quality of work life (42–44). In this regard, the improvement of working processes involving productivity, quality, service delivery, safety, employee morale, and cost control could improve healthcare work environment standards (45–48).

## Limitations

The present study is not without limitations. First, uncontrollable factors apart from the study variables, such as daily schedules and personal habits, may affect the nurse’s QWL. Second, it is important to apply caution when extrapolating the results of this study because the sample was not chosen at random but rather using a convenience sampling approach. Given that the study was cross-sectional in nature, it was difficult to draw conclusions regarding cause-and-effect relationships. The sample was limited to CCU nurses only; therefore, it appears that there were some limitations on sample selection. Also, due to sampling bias, many different nurses might have been overlooked as a result.

## Conclusion

The aim of the current study was to examine QNWL and its influencing factors among Jordanian nurses working in CCUs. According to the study’s findings, the majority of Jordanian nurses working in CCUs reported having a fair QNWL. Additionally, the study reveals that QNLW can be promoted by good-quality sleep and a peaceful working environment. Therefore, in order to raise the quality of life for nurses, organizations and administrators should pay attention to these factors.

## Data availability statement

The original contributions presented in the study are included in the article/supplementary material, further inquiries can be directed to the corresponding author.

## Ethics statement

The studies involving humans were approved by The Jordanian Al-Zaytoonah University gave the approval for the study with IRB No. (1269312080). The studies were conducted in accordance with the local legislation and institutional requirements. Written informed consent for participation in this study was provided by the participants’ legal guardians/next of kin. Written informed consent was obtained from the individual(s), and minor(s)’ legal guardian/next of kin, for the publication of any potentially identifiable images or data included in this article.

## Author contributions

MA: Conceptualization, Writing – original draft, Writing – review & editing. SA-M: Methodology, Validation, Writing – original draft, Writing – review & editing. KA-M: Supervision, Writing – original draft, Writing – review & editing. IO: Conceptualization, Writing – original draft, Writing – review & editing. GA: Writing – original draft, Writing – review & editing. AS: Writing – original draft, Writing – review & editing. SA: Resources, Writing – original draft. SF: Writing – original draft, Writing – review & editing.

## References

[1] Rastegari M Khani A Ghalriz P Eslamian J. Evaluation of quality of working life and its association with job performance of the nurses. Iran J Nurs Midwifery Res. (2010) 15:224–8. PMID: 22049285 PMC3203281

[2] Moradi T Maghaminejad F Azizi-Fini I. Quality of working life of nurses and its related factors. Nurs Midwifery Stud. (2014) 3. doi: 10.5812/nms.19450 PMC4228533 25414904

[3] Swamy DR Nanjundeswaraswamy TS Rashmi S. Quality of work life: scale development and validation. Int J Caring Sci. (2015) 8:281. Available at: https://www.internationaljournalofcaringsciences.org

[4] Hemanathan R Sreelekha PP Golda M. Quality of work life among nurses in a tertiary care hospital. Health Car. (2017) 5:1–8. doi: 10.19080/JOJNHC.2017.05.555667

[5] Raeissi P Rajabi MR Ahmadizadeh E Rajabkhah K Kakemam E. Quality of work life and factors associated with it among nurses in public hospitals, Iran. J Egypt Public Health Assoc. (2019) 94:1–8. doi: 10.1186/s42506-019-0029-2 32813080 PMC7364675

[6] Brooks BA Anderson MA. Defining quality of nursing work life. Nurs Econ. (2005) 23:319–26. PMID: 16459904

[7] Ramesh N Nisha C Josephine AM Thomas S Joseph B. A study on quality of work life among nurses in a medical college hospital in Bangalore. Natl J Community Med. (2013) 4:471–4.

[8] Devi B Hajamohideen O. A study on quality of work life among nurses working in private hospitals an Thanjavur, Tamilnadu. IOSR Int J Bus Manag. (2018) 20:61–3. doi: 10.9790/487X-2004026163

[9] Nair GS. A study on the effect of quality of work life (QWL) on organisational citizenship behaviour (OCB)-with special reference to college teachers is Thrissur district, Kerala. Integral Rev. (2013) 6:34–46. Available at: https://www.intergraluniversity.ac.in/net/journals

[10] Brooks BA Storfjell J Omoike O Ohlson S Stemler I Shaver J. Assessing the quality of nursing work life. Nurs Adm Q. (2007) 31:152–7. doi: 10.1097/01.NAQ.0000264864.94958.8e 17413509

[11] Ibrahim NK Alzahrani NA Batwie AA Abushal RA Almogati GG Sattam MA. Quality of life, job satisfaction and their related factors among nurses working in king Abdulaziz university hospital, Jeddah, Saudi Arabia. Contemp Nurse. (2016) 52:486–98. doi: 10.1080/10376178.2016.1224123, PMID: 27586128

[12] Panahi D Pirposhteh EA Moradi B Poursadeqiyan M Sahlabadi AS Kavousi A. Effectiveness of educational intervention on reducing oxidative stress caused by occupational stress in nurses: a health promotion approach. J Edu Health Promot. (2022) 11:1–8. doi: 10.4103/jehp.jehp_1425_21 PMC9621363 36325207

[13] Janaabadi H. Two effective factors in the staffs performance: quality of life and quality of working life. Zahedan J Res Med Sci. (2012) 13:9.

[14] Alharbi MF Alahmadi BA Alali M Alsaedi S. Quality of nursing work life among hospital nurses in Saudi Arabia: A cross-sectional study. J Nurs Manag. (2019) 27:1722–30. doi: 10.1111/jonm.12863, PMID: 31495010

[15] Jaber HJ Abu Shosha GM Al-Kalaldeh MT Oweidat IA Al-Mugheed K Alsenany SA. Perceived relationship between horizontal violence and patient safety culture among nurses. Risk Manag Healthcare Policy. (2023) 1545–53. doi: 10.2147/RMHP.S419309 PMC10438459 37602363

[16] Oweidat I Al-Mugheed K Alsenany SA Abdelaliem SMF Alzoubi MM. Awareness of reporting practices and barriers to incident reporting among nurses. BMC Nurs. (2023) 22:231. doi: 10.1186/s12912-023-01376-9 37400810 PMC10318788

[17] Al-Mugheed K Farghaly SM Baghdadi NA Oweidat I Alzoubi MM. Incidence, knowledge, attitude and practice toward needle stick injury among nursing students in Saudi Arabia. Front Public Health. (2023) 11:1160680. doi: 10.3389/fpubh.2023.1160680 37213613 PMC10192570

[18] Suleiman K Hijazi Z Al Kalaldeh M Sharour LA. Quality of nursing work life and related factors among emergency nurses in Jordan. J Occup Health. (2019) 61:398–406. doi: 10.1002/1348-9585.12068, PMID: 31215754 PMC6718837

[19] Mohammadi A Sarhanggi FO Ebadi AB Daneshmandi MO Reiisifar AF Amiri FA. Relationship between psychological problems and quality of work life of intensive care unit nurses. Iran J Crit Care Nurs. (2011) 4:135–40.

[20] Barrientos-Trigo S Vega-Vázquez L De D-CR Badanta-Romero B Porcel-Gálvez AM. Interventions to improve working conditions of nursing staff in acute care hospitals: scoping review. J Nurs Manag. (2018) 26:94–107. doi: 10.1111/jonm.12538, PMID: 29327478

[21] Almalki MJ FitzGerald G Clark M. Quality of work life among primary health care nurses in the Jazan region, Saudi Arabia: a cross-sectional study. Hum Resour Health. (2012) 10:1–13. doi: 10.1186/1478-4491-10-30 22971150 PMC3543175

[22] Eslamian J Akbarpoor AA Hoseini SA. Quality of work life and its association with workplace violence of the nurses in emergency departments. Iran J Nurs Midwifery Res. (2015) 20:56–62. PMID: 25709691 PMC4325414

[23] Faghihi M Farshad A Abhari MB Azadi N Mansourian M. The components of workplace violence against nurses from the perspective of women working in a hospital in Tehran: a qualitative study. BMC Womens Health. (2021) 21:1–13. doi: 10.1186/s12905-021-01342-0 34011330 PMC8136170

[24] Dehghan-Chaloshtari S Ghodousi A. Factors and characteristics of workplace violence against nurses: a study in Iran. J Interpers Violence. (2020) 35:496–509. doi: 10.1177/0886260516683175, PMID: 29294631

[25] Lebni JY Toghroli R Abbas J Kianipour N NeJhaddadgar N Salahshoor MR. Nurses’ work-related quality of life and its influencing demographic factors at a public hospital in western Iran: a cross-sectional study. Int Q Community Health Educ. (2021) 42:37–45. doi: 10.1177/0272684X20972838, PMID: 33201756

[26] Vatani J Arami M Khanikosarkhizi Z Shahabi Rabori MA Khandan M Dehghan N. Safety climate and related factors in rehabilitation nurses of hospitals in Iran. Work. (2021) 68:189–96. doi: 10.3233/WOR-203368, PMID: 33427720

[27] Baysal HY Yildiz M. Nursing’s job life quality’s effect on job satisfaction. Intern J Caring Sci. (2019) 12:1056. Available at: https://www.internationaljournalofcaringsciences.org

[28] Suleiman KH Yates BC Berger AM Pozehl B Meza J. Translating the Pittsburgh sleep quality index into Arabic. West J Nurs Res. (2010) 32:250–68. doi: 10.1177/0193945909348230, PMID: 19915205

[29] Andersson IS Lindgren M. The Karen instruments for measuring quality of nursing care. Item Analysis Vård I Norden. (2008) 28:14–8. doi: 10.1177/010740830802800

[30] Lindgren M Andersson IS. The Karen instruments for measuring quality of nursing care: construct validity and internal consistency. Int J Qual Health Care. (2011) 23:292–301. doi: 10.1093/intqhc/mzq092, PMID: 21242159

[31] Malak MZ Abu Safieh AM. Association between work-related psychological empowerment and quality of nursing care among critical care nurses. J Nurs Manag. (2022) 30:2015–22. doi: 10.1111/jonm.13641, PMID: 35478472

[32] Nayeri ND Salehi T Noghabi AAA. Quality of work life and productivity among Iranian nurses. Contemp Nurse. (2011) 39:106–18. doi: 10.5172/conu.2011.39.1.106 21955271

[33] Lee YW Dai YT McCreary LL. Quality of work life as a predictor of nurses' intention to leave units, organisations and the profession. J Nurs Manag. (2015) 23:521–31. doi: 10.1111/jonm.12166, PMID: 24238014

[34] Hesam M Asayesh H Roohi G Shariati A Nasiry H. Assessing the relationship between nurses' quality of work life and their intention to leave the nursing profession. Q J Nurs Manag. (2012) 1:28–36.

[35] Almalki MJ FitzGerald G Clark M. The relationship between quality of work life and turnover intention of primary health care nurses in Saudi Arabia. BMC Health Serv Res. (2012) 12:1–11. doi: 10.1186/1472-6963-12-314 22970764 PMC3507760

[36] Dargahi H Gharib MI Goodarzi M. Quality of work life in nursing employees of Tehran University of Medical Sciences hospitals. HAYAT. (2007) 13:13–21.

[37] Dehghannyieri N Salehi T Asadinoghabi AA. Assessing the quality of work life, productivity of nurses and their relationship. Iran J Nurs Res. (2008) 3:27–37.

[38] Sohrabi Y Yarmohammadi H Pouya AB Arefi MF Poursadeqiyan M. Prevalence of job burnout in Iranian nurses: A systematic review and Meta-analysis. Work. (2022) 73:937. doi: 10.3233/WOR-210283 35988237

[39] Nayeri ND Negarandeh R Vaismoradi M Ahmadi F Faghihzadeh S. Burnout and productivity among Iranian nurses. Nurs Health Sci. (2009) 11:263–70. doi: 10.1111/j.1442-2018.2009.00449.x, PMID: 19689634

[40] Fu X Xu J Song LI Li H Wang J Wu X. Validation of the Chinese version of the quality of nursing work life scale. PLoS One. (2015) 10:e0121150. doi: 10.1371/journal.pone.0121150, PMID: 25950838 PMC4423946

[41] Momeni B Shafipour V Esmaeili R Yazdani CJ. The relationship between the quality of work life and sleep in nurses at the intensive care units of teaching hospitals in Mazandaran. Iran J Nurs Midwifery Sci. (2016) 3:28–34.

[42] De Lira CR Akutsu RD Costa PR Leite LD da Silva KB Botelho RB. Occupational risks in hospitals, quality of life, and quality of work life: A systematic review. Int J Environ Res Public Health. (2021) 18:11434. doi: 10.3390/ijerph182111434 34769950 PMC8582940

[43] Abbasi M Zakerian A Mehri A Poursadeghiyan M Dinarvand N Akbarzadeh A. Investigation into effects of work-related quality of life and some related factors on cognitive failures among nurses. Int J Occup Saf Ergon. (2017) 23:386–92. doi: 10.1080/10803548.2016.1216991, PMID: 27599912

[44] Khaleghi S Sadeghimoghadam A Moradi Y Jafarizadeh H Ghalavand M Poursadeqiyan M. Et Al is Nurses' job satisfaction related to occupational health and safety management? Iran J Public Health. (2021) 50:1738–9. doi: 10.18502/ijph.v50i8.6841, PMID: 34917550 PMC8643524

[45] Alzoubi MM KS H AM R al-Zoubi KM al-Mugheed K Alsenany SA. Effect of total quality management intervention on nurse commitment and nurse performance: A quasi-experimental study. Medicine. (2023) 102:e35390. doi: 10.1097/MD.0000000000035390 37800832 PMC10552992

[46] Al-Mugheed K Bayraktar N. Patient safety attitudes among critical care nurses: A case study in North Cyprus. Int J Health Plann Manag. (2020) 35:910–21. doi: 10.1002/hpm.2976, PMID: 32329530

[47] Oweidat I Omari A Albashtawy M Saleh AO Alrahbeni T al-Mugheed K. Factors affecting the quality of working life among nurses caring for Syrian refugee camps in Jordan. Hum Resour Health. (2024) 22:1. doi: 10.1186/s12960-023-00884-8 38167317 PMC10763280

[48] Abbasi M Zakerian A Akbarzade A Dinarvand N Ghaljahi M Poursadeghiyan M. Investigation of the relationship between work ability and work-related quality of life in nurses. Iran J Public Health. (2017) 46:1404–12. PMID: 29308385 PMC5750353

